# Famine food of vegetal origin consumed in the Netherlands during World War II

**DOI:** 10.1186/s13002-017-0190-7

**Published:** 2017-11-17

**Authors:** Tom Vorstenbosch, Ingrid de Zwarte, Leni Duistermaat, Tinde van Andel

**Affiliations:** 1 Institute of Biology, Leiden University, P.O. Box 9505, 2300 RA Leiden, the Netherlands; 2 University of Amsterdam/NIOD Institute for War, Holocaust and Genocide Studies, Herengracht 380, 1016 CJ Amsterdam, the Netherlands; 3 Naturalis Biodiversity Center, PO Box 9517, 2300 RA Leiden, the Netherlands; 4 Wageningen University, Biosystematics Group, Droevendaalsesteeg 1, 6708 BP Wageningen, the Netherlands

**Keywords:** Dutch famine, Emergency food, Recipes, Tulip bulbs, Wild plant collection, World War II

## Abstract

**Background:**

Periods of extreme food shortages during war force people to eat food that they normally do not consider edible. The last time that countries in Western Europe experienced severe scarcities was during World War II. The so-called Dutch famine or Hunger Winter (1944–1945) made at least 25,000 victims. The Dutch government took action by opening soup kitchens and providing information on wild plants and other famine food sources in “wartime cookbooks.” The Dutch wartime diet has never been examined from an ethnobotanical perspective.

**Methods:**

We interviewed 78 elderly Dutch citizens to verify what they remembered of the consumption of vegetal and fungal famine food during World War II by them and their close surroundings. We asked whether they experienced any adverse effects from consuming famine food plants and how they knew they were edible. We identified plant species mentioned during interviews by their local Dutch names and illustrated field guides and floras. We hypothesized that people living in rural areas consumed more wild species than urban people. A Welch *t* test was performed to verify whether the number of wild and cultivated species differed between urban and rural citizens.

**Results:**

A total number of 38 emergency food species (14 cultivated and 21 wild plants, three wild fungi) were mentioned during interviews. Sugar beets, tulip bulbs, and potato peels were most frequently consumed. Regularly eaten wild species were common nettle, blackberry, and beechnuts. Almost one third of our interviewees explicitly described to have experienced extreme hunger during the war. People from rural areas listed significantly more wild species than urban people. The number of cultivated species consumed by both groups was similar. Negative effects were limited to sore throats and stomachache from the consumption of sugar beets and tulip bulbs. Knowledge on the edibility of famine food was obtained largely by oral transmission; few people remembered the written recipes in wartime cookbooks.

**Conclusion:**

This research shows that 71 years after the Second World War, knowledge on famine food species, once crucial for people’s survival, is still present in the Dutch society. The information on famine food sources supplied by several institutions was not distributed widely. For the necessary revival of famine food knowledge during the 1940s, people needed to consult a small group of elders. Presumed toxicity was a major reason given by our participants to explain why they did not collect wild plants or mushrooms during the war.

## Background

Famine has been part of human history since the foundation of agriculture. During periods of severe hunger, people resort to unconventional food that they do not or hardly eat in “normal” times, so-called famine or emergency’ foods [1, 2]. Generally, this means plants, animals, and mushrooms collected from the wild and repulsive or unfamiliar food that is normally not considered suitable for human consumption, such as fodder and vegetable waste [3–5]. A revert to famine foods, however, implies that knowledge on wild or otherwise unconventional edible species is still present in the community. In industrialized, Western European societies, where people have become less reliant on their natural surroundings for the past century, this may pose a problem. The last time that Western Europe had to cope with extreme food shortages was in the Second World War [6]. This was particularly severe in certain parts of the Netherlands, an urbanized country with relatively little natural vegetation and a high percentage of agricultural grounds. While foraging for wild food was still common in eastern and southern Europe around the 1940s [7–9], it had long been abandoned in the Netherlands.

In the winter and early spring of 1944–1945, food shortages were so severe that the period is known as the Dutch famine or Hunger Winter [10, 11]. During the military operation “Market Garden,” the allied troops had liberated the southern Dutch provinces, but they failed to advance towards Arnhem and cross the Rhine River. This left the northeastern Dutch provinces occupied till April 1945 and the northwestern until in the beginning of May 1945 [12, 13]. Aggravated by a simultaneously initiated Dutch railway strike on 17 September 1944 and a temporary German embargo on inland shipping that lasted several weeks, food in the densely populated western parts of the Netherlands became scarcer. However, it is hard to point out a single causing event for the famine. A complex accumulation of various events altogether led to this hunger. The extreme fuel shortages after the only domestic mining area was liberated the summer before, the rise of clandestine trade and production, the relatively severe frost from late December 1944 until the end of January 1945, and the seizing of scant resources of vegetables, fruit, cereals, fat, and livestock all worsened the situation for the urban Dutch citizens [14, 15]. The Hunger Winter made approximately 25,000 victims, mostly elder males, comparable to the Greek famine of 1941–1944 [16, 17]. The real number of victims as result of the Dutch food crisis was likely higher, as many diseases broke out due to the general lack of nutrition [18]. Consequences of prenatal exposure to malnutrition are still visible in adult health status today [19–29].

**Fig. 1:**
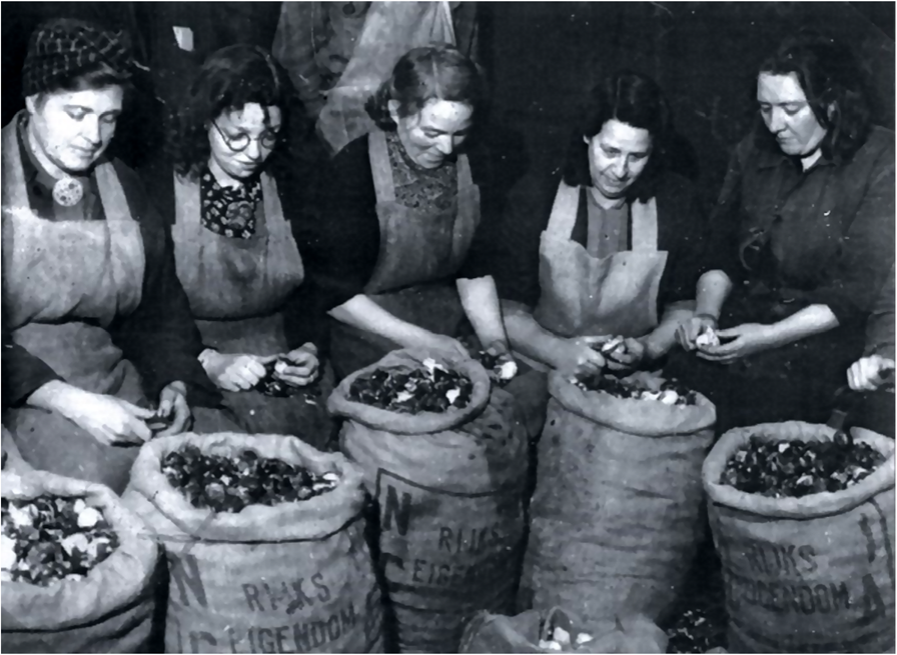
Women preparing tulip bulbs for a soup kitchen in Rotterdam. Source: [85]

Based on the experiences with food coupons during the First World War, the Dutch government distributed provision in the form of ration slips to be exchanged for food, clothing, and other products. Initially in October 1944, the rations had a caloric value of 1400 per day, but this dropped to 500 kcal in January 1945 [30]. For many Dutch citizens, the Hunger Winter is inseparably associated with tulip bulbs and sugar beets (Fig. 1).

Normally used for sugar production, sugar beets were distributed in early 1945 under this food distribution system and processed with tulip bulbs and potato peels in soup kitchens to feed severely exhausted citizens [31, 32]. Despite the fact that this system saved a substantial number of lives, it was not airtight in distributing the scarce food resources to those in highest need. Over 40% of the agricultural production disappeared into non-documented or illegal circuits, leaving less food to people without connections or the money to buy or barter food on the black market [33–35].

Collecting wild food was another way to complement the official food distribution system. Wild plants and mushrooms provide a welcome source of micronutrients in times where cultivated crops are scarce and food is unvarying [36]. Anecdotal evidence exists from letters and diaries on wild collection during the World War II by people who normally did not do this [37, 38].

**Fig. 2:**
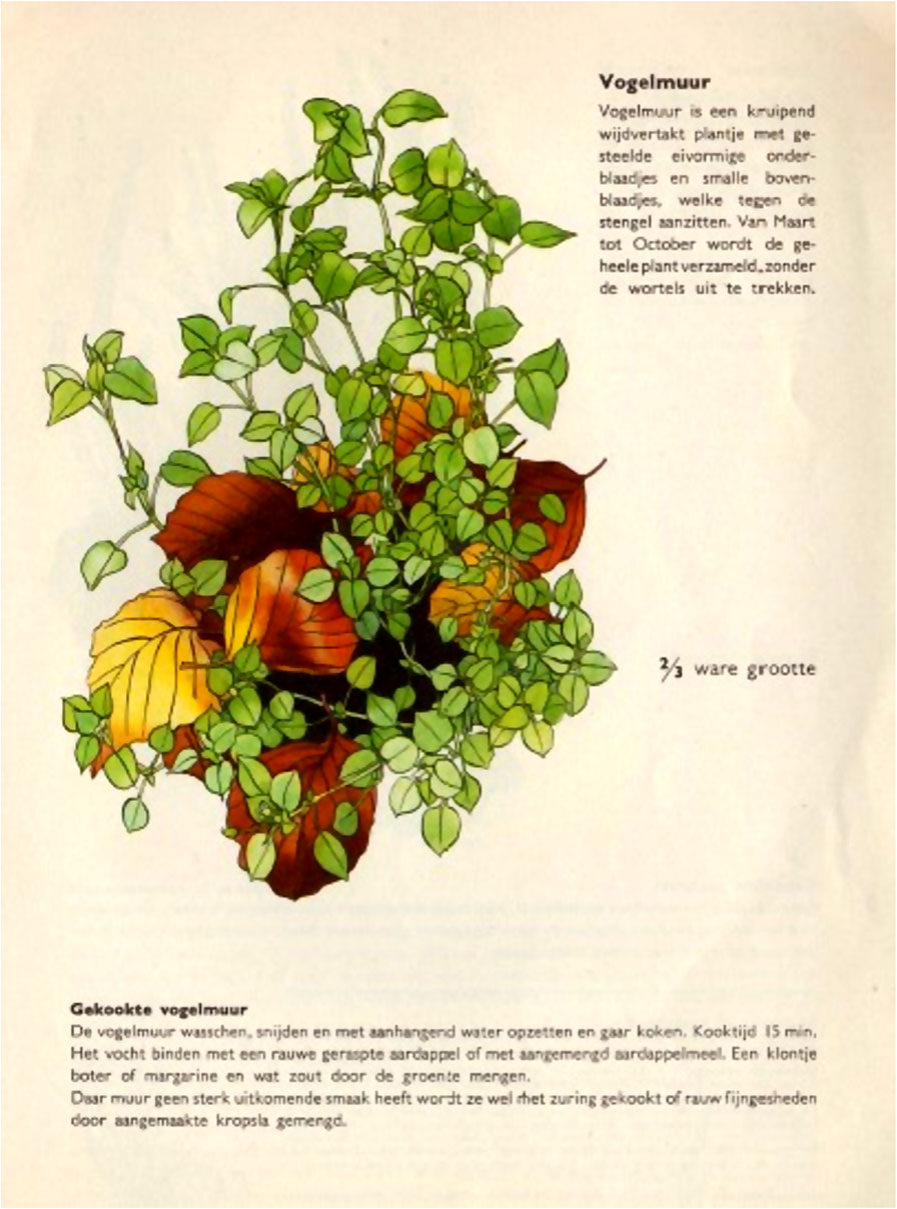
Government leaflet on wild collection and preparation of common chickweed [40]

During previous conflicts and their accompanying shortages, like in Germany during the First World War, programmes were set up to inform the public about dealing with famine and to suggest alternative food sources [39]. The Dutch government distributed illustrated folders [40] on the collection and preparation of wild plants and mushrooms, such as common nettle (*Urtica dioica* L.), common chickweed (*Stellaria media* (L.) Vill.), and sorrel (*Rumex* spp.) (Fig. 2). These booklets suggest that at least some Dutch were knowledgeable about their natural environment as a resource for food. Whether these booklets reached large parts of the Dutch society remains unknown.

No previous ethnobotanical research exits on the collection and consumption of wild plant species or unconventional crops as emergency food by Dutch citizens during World War II. People living in rural areas generally have more knowledge on edible wild food than urban citizens [36, 41]. However, during 1940–1945, the need to collect wild food must have been higher among urban people, as they had no vegetable gardens or livestock as means of subsistence.

The aim of this study was to find out whether survivors of the Dutch famine still remember the species of famine food consumed by themselves or people in their near surroundings (family, friends, neighbors) in periods of severe hunger throughout the war. Our research questions were the following: (1) Which wild plants and mushrooms or other cultivated crops not primarily meant for human consumption were eaten as famine food in the period 1940–1945?; (2) Did people use the wartime cookbooks and pamphlets for preparing food with unfamiliar and/or wild collected ingredients?; and (3) Did people living in rural areas during 1940–1945 consume more species of wild plants, mushrooms, or cultivated famine food than people living in urban areas?; Do war survivors remember any adverse effects of the consumption of famine food?

We hypothesized that people who lived in rural areas during World War II consumed more species of wild food plants or mushrooms than those who spent the war years in urban areas, as the latter were less knowledgeable on these species and had limited access to nature areas to collect them.

## Methods

### Data collection

We defined famine food as species of vegetal and fungal source, collected in the wild, waste material of edible crops, fodder, and other cultivated crops not meant for (direct) human consumption, like ornamental plants. The species of famine food should have been collected predominantly during the war to be categorized as emergency food. We constructed a preliminary list of emergency food plants (Table 1) using documentation on food distribution, recipe pamphlets, and wartime cookbooks, digitized letters, and diaries archived in the library of the Netherlands Institute for War, Holocaust and Genocide Studies [17, 42–57]. Local names, distribution, and prevalence of plant species in the 1940s were checked with recent and historic floristic literature [58, 59].

**Table 1.** List of famine food species from published literature, unpublished personal diaries, and letters, pamphlets, and wartime cookbooks archived in the library of the Dutch National Institute of War Documentation (NIOD), personal war collections, and online sources, with scientific name, English and Dutch name, family, and distribution in 1942

Scientific name	English name	Dutch name	Family	Distribution around 1942 [47]	Source(s)
*Aegopodium podagraria* L.^a^	Ground elder	Zevenblad	*Apiaceae*	Common	–
*Aesculus hippocastanum* L.^a^	Horse chestnut	Wilde kastanje	*Sapindaceae*	Cultivated	–
*Agaricus campestris*	Field mushroom	Gewone weidechampignon	*Agaricaceae*	–	[45]
*Atriplex hortensis* L.	Garden orache	Tuinmelde	*Amaranthaceae*	Cultivated and wild	[40, 48]
*Bellis perennis* L.^a^	Common daisy	Madeliefje	*Asteraceae*	Very common	–
*Beta vulgaris* L. subsp. *vulgaris* var. *altissima* ^a^	Sugar beet	Suikerbiet	*Brassicaceae*	Cultivated	[17, 52–54]
*Betula* ssp*.* L.^a^	Birch (leaves)	Berk	*Betulaceae*	Common	[42]
*Boletus edulis*	Porcini	Eekhoorntjesbrood	*Boletaceae*	–	[45]
*Brassica oleracea* L. convar. *botrytis* var. *botrytis*	Cauliflower (foliage)	Bloemkoolblad	*Brassicaceae*	Cultivated	[48, 51]
*Cantharellus cibarius*	Chanterelle	Cantharellen	*Cantharellaceae*	–	[40, 42, 45]
*Castanea sativa* Mill.^a^	Sweet chestnut	Tamme kastanje	*Fagaceae*	–	[40, 42, 45, 55]
*Cichorium intybus* L. var. *sativum* ^*a*^	Chicory	Koffiecichorei	*Asteraceae*	Fairly common in river areas, elsewhere escaped from cultivation	[17, 45]
*Coryllus avellana* L.	Hazelnut	Hazelnoot	*Betulaceae*	Common, sometimes cultivated	[43, 45]
*Crataegus* sp. Tourn. *ex* L.^a^	Hawthorn	Meidoorn	*Rosaceae*	*C*. *monogyna*, common, *C*. *oxyacantha*, fairly rare	[42]
*Crocus* ssp. L.^a^	Crocus	Krokus	*Iridaceae*	Cultivated	[17]
*Dahlia* ssp. Cav.^a^	Dahlia	Dahlia	*Asteraceae*	Cultivated	[47]
*Daucus carota* L. subsp. *sativus* ^a^	Carrot (foliage)	Wortel (loof)	*Apiaceae*	Cultivated and common	[42, 45, 48, 51]
*Fagus sylvatica* L.^a^	European beech	Beuk	*Fagaceae*	Common in the southeast	[42, 45]
*Fragaria vesca* L.	Woodland strawberry	Wilde aardbei	*Rosaceae*	Common	[45]
*Galium aparine* L.^a^	Cleavers	Kleefkruid	*Rubiaceae*	Common	–
*Galium odoratum* L.^a^	Sweetcented woodruff	Lievevrouwebedstro	*Rubiaceae*	Common in the extreme south	[41]
*Gladiolus* ssp. L.^a^	Gladiola	Gladiool	*Iridaceae*	Cultivated	[17, 56, 57]
*Glechoma hederacea* L.^a^	Ground-ivy	Hondsdraf	*Lamiaceae*	Very common	–
*Helianthus tuberosus* L.^b^	Jerusalem artichoke	Aardpeer	*Asteraceae*	Cultivated	[17]
*Hyacinthus orientalis* L.^a^	Common hyacinth	Hyacint	*Asparagaceae*	Cultivated	[17]
*Iris* ssp. L.^a^	Iris	Iris	*Iridaceae*	Cultivated	[17]
*Juglans regia* L.^a^	English walnut	Walnoot	*Juglandaceae*	Often cultivated	[17, 40, 43, 45]
*Lamium* sp. L.	Dead-nettle	Dovenetel	*Lamiaceae*	Common	[51]
*Limonium vulgare* Mill.	Common sea lavender	Lamsoor	*Plumbaginaceae*	Fairly common; coastal areas	[39]
*Malus baccata* (L.) Borkh.^a^	Wild apple	Kersappel	*Rosaceae*	Cultivated	[40]
*Malus domestica* Borkh.	Apple (kernel/peels)	Appel	*Rosaceae*	Cultivated	[42, 45, 46, 57, 87]
*Malus floribunda* Siebold ex. Van Houtte	Japanese crabapple	Japanse sierappel	*Rosaceae*	Cultivated	[39]
*Petasites hybridus* ( L.) G.Gaertn., B.Mey. & Scherb.^a^	Butterbur	Groot hoefblad	*Asteraceae*	Fairly common	–
*Plantago lanceolata* L.^a^	English plantain	Smalle weegbree	*Plantaginaceae*	Very common	–
*Plantago major* L.^a^	Broadleaf plantain	Brede weegbree	*Plantaginaceae*	Very common	–
*Portulaca oleracea* L.^a^/*Claytonia perfoliata* Donn ex. Willd	Summer purslane/winter purslane	Postelein	*Portulacaceae*/*montiaceae*	Cultivated, sometimes abundant	[46, 87]
*Prunus avium* L.	Cherry (fruit stems)	Kersen	*Rosaceae*	Cultivated	[42]
*Quercus robur* L.	English oak	Eik (eikels)	*Fagaceae*	Common; also cultivated	[86]
*Raphanus sativus* L.	Radish (foliage)	Radijs (loof)	*Brassicaceae*	Cultivated	[48]
*Raphanus sativus* L. subsp. *niger* ^b^	Black radish	Rammenas	*Brassicaceae*	Cultivated	–
*Ribes nigrum* L.	Blackcurrant	Zwarte bes	*Grossulariaceae*	Culivated and wild	[17, 50]
*Ribes rubrum* L.	Redcurrant	Aalbes	*Grossulariaceae*	Culivated and wild; mostly in the south	[45]
*Ribes uva-crispa* L.	Gooseberry	Kruisbes	*Grossulariaceae*	Cultivated and wild	[43]
*Rosa* ssp. L.	Rose (hips)	Rozenbottel	*Rosaceae*	Common	[42, 45, 50]
*Rubus* ssp. L.	Blackberry	Braam	*Rosaceae*	Common	[40, 42, 43, 45]
*Rumex acetosa* L./*Rumex crispus* L.	Common sorrel/curly dock	Veldzuring/krulzuring	*Polygonaceae*	Very common on grasslands/common on fertile grounds	[17, 40, 42, 45]
*Salicornia europaea* L.	Common glasswort	Zeekraal	*Amaranthaceae*	Common; coastal areas	[40, 45]
*Sambucus nigra* L.	Black elder	Vlier	*adoxaceae*	Common; also in dunes	[42, 45]
*Stellaria media* (L.) Vill.	Common chickweed	Vogelmuur	*Caryophyllaceae*	Very common in grassland and open grounds	[40, 51]
*Taraxacum officinale* L.	Common dandelion	Paardenbloem	*Asteraceae*	Common	[40, 45, 51]
*Tilia* ssp. L.	Linden (blossom)	Lindebloesem	*Malvaceae*	Cultivated and wild	[17, 42, 43, 45, 87]
*Trifolium* ssp. L.	Clover	Klaver	*Fabaceae*	Very common	-
*Tulipa* ssp. L.	Tulip	Tulp	*Liliaceae*	Cultivated	[17, 56, 85]
*Typha latifolia* L.	Broadleaf cattail	Lisdodde	*Typhaceae*	Common	–
*Urtica dioica* L.	Common nettle	Brandnetel	*Urticaceae*	Very common	[40, 45, 48, 51]
*Vaccinium myrtylis*	European blueberry	Bosbes	*Ericaceae*	Common in forests	[40, 45]
*Vaccinium oxycoccus* Hill	Cranberry	Veenbes	*Ericaceae*	Rare	[40]
*Vaccinium vitis-idea* L.	Cowberry	Vossenbes	*Ericaceae*	Fairly common, rare in the west	[40, 45]
*Valerianella locusta* L. DC.	Common cornsalad	Veldsla	*Caprifoliaceae*	Common	[87]

^a^Species on list discussed with participants

^b^Excluded from results as species were not wild harvested but sold commercially during the Second World War

We conducted interviews with World War II survivors, their descendants, and close relatives from February to April 2016. We pre-tested the questionnaires among retired botanists working as honorary staff at the Naturalis herbarium in Leiden (L). After this test phase, requests for performing interviews were sent out to several elderly homes. Most interviews were held among inhabitants of elderly homes in the major cities in the western Netherlands. Participants had to remember at least one species of emergency food consumed during 1940–1945 to be included in our analysis. The questionnaires started with inquiries on people’s age, residence during the war, whether they experienced severe hunger during this period, and whether they were familiar with wartime cookbooks and pamphlets. The questionnaire continued with a free-listing exercise to name as many plant or mushroom species that were eaten by themselves or by others in their close surroundings and that were not consumed outside wartime. Other questions included: If you ate things like sugar beets and tulip bulbs, how did you or your relatives know these were edible? Do you remember if the consumption of emergency food resulted in uneasiness or illness?

Finally, we showed the participants our preliminary list of emergency food species to verify whether they remembered to have eaten any of those during the war. As participants mostly mentioned plants by their common Dutch names, we used illustrated field guides of the Dutch flora to assist the participants in clarifying the species they had consumed and to verify scientific names [60, 61]. Only emergency foods that could be linked to a specific genus or species were included in our quantitative analysis. Famine food described as “flower bulbs” and “mushrooms” were noted, but excluded from our quantitative analysis.

**Fig. 3:**
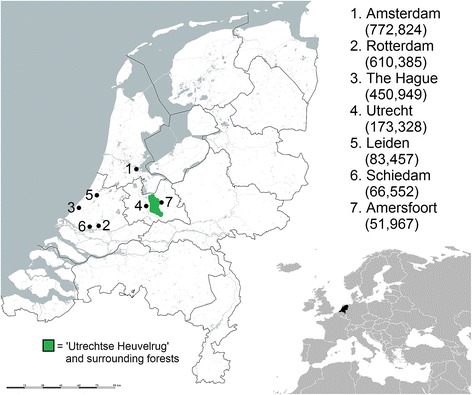
Population numbers on 1 January 1945 of the major cities in the western Netherlands. Source [63]

Apart from face-to-face interviews, we emailed questionnaires to amateur historical discussion groups throughout the Netherlands. Questionnaires were also handed out in the city of Leiden during a meeting of the historic association “Oud Leiden”. We created an online survey tool by using Surveymonkey Inc. [62] and posted the link to this survey on Facebook pages of Dutch historical societies and war museums. The face-to-face questionnaires, printed forms, and online survey consisted of exactly the same questions, so they could be processed in a similar way. The requests to elderly homes led to face-to-face interview sessions in the cities Amsterdam, Rotterdam, The Hague, the major cities located in the west of the country, and Utrecht, in the center of the Netherlands (Fig. 3). The wartime conditions in these cities were the most severe, due to their population density, which made it more likely that people ate famine food [63]. Finally, the first author gave a public lecture at Naturalis Biodiversity Center to present his preliminary findings. He invited several interview participants and other wartime survivors. After the lecture, group discussions were held on the identification of freshly collected emergency food specimens and flower bulbs purchased at a tourist market in Amsterdam.

### Data analysis

To test whether people from rural areas had more knowledge on edible wild plants and mushrooms than people who had spent the war in the city, we compared the number of wild plant species mentioned by the two groups. The urban group included people that lived between 1940 and 1945 in municipalities with a population over 50,000, as was the case in Amsterdam, Rotterdam, The Hague, Schiedam, and Leiden (Fig. 3). Population numbers for 1 January 1945 were taken as reference [63]. The rural group consisted of people living during the war in municipalities with a population under 50,000, in addition to Amersfoort and Utrecht. The east of Utrecht borders a large forested area called the “Utrechtse Heuvelrug” (Utrecht hills), while the other larger western cities lack extensive woody vegetation close by [64]. For this reason and the reports of people collecting in these forests during the war [37], Utrecht citizens were placed in the rural group. The same accounts for Amersfoort, located on the other side of the Utrechtse Heuvelrug.

The number of wild and cultivated species was scored for every interviewee. When a species was cultivated as well as growing in the wild, as is the case with English walnut (*Juglans regia* L.) or blackberry (*Rubus* spp*.*), it was verified whether the collecting related to wild individuals (for only these were considered as emergency foods). To calculate citation frequency, we counted the number and percentage of interviews in which a specific species was mentioned. We did not take into account how many times a certain species was mentioned within a single interview. We separately analyzed the most frequently mentioned species during the free-listing exercise only and calculated a cognitive salience index [65], which ranges from 0 (never mentioned) to 1 (mentioned by all participants) and calculated as follows:

$$ S=F/\left(N\mathrm{mP}\right) $$

in which *S* = salience index, *F* = frequency, mP = mean position of mentioning, *N* = total number of participants.

A Welch *t* test (one-tailed) was applied three times on these data under the assumption that the two groups had unequal variances. Differences were considered significant when *p* values were smaller than 0.05. The statistical analysis was performed with R studio version 0.98.1091 [66]. The first test verified whether there was a significant difference in the number of wild species eaten by people from rural or urban areas. The second tested whether there was a difference in number of cultivated famine food species eaten by rural vs. urban groups. The third test was applied to see whether there was a difference in the overall number of famine food species consumed by people from rural and urban areas.

## Results

A total of 78 interviews were completed (41 face-to-face interviews, 13 digital questionnaires from our online survey, 19 hand-filled questionnaires sent by post, three by e-mail, and two telephone interviews). Four incomplete online-survey responses were discarded. Of our urban group (*n* = 52), nine persons or their parents lived in Rotterdam, The Hague (23), Leiden (13), Amsterdam (6), and Schiedam (1) at the time of the liberation in 1945. Our rural group (*n* = 26) consisted of six persons from Utrecht, one from Amersfoort and 19 persons that lived in other municipalities with less than 50,000 people on the 1 of January in 1945. The average age of the participants at the time of liberation was 12, with birth dates of the interviewees between 1910 and 1947. Three interviews were completely “second-hand,” as participants were either very young during the war or born afterwards, but remembered stories from their parents on famine food consumption during the war. Not all war survivors experienced similar food shortages: 24 interviewees explicitly described extreme hunger, while 14 people said they did not remember to have suffered from severe hunger. Four of these latter claims, however, were highly contradictory to their stories that followed the question on personal experience of hunger during the war (e.g., “having to go to bed often without any food” or “living on one sandwich a day”.

The 78 interviews resulted in a list of 14 cultivated species (including four fodder species, six ornamental species, and three species of waste material), 21 wild plant species, and three wild fungal species. The complete list of emergency food species mentioned during our interview is listed with scientific and author names, families, vernacular names in Dutch and specific uses in Table 2. Emergency food species include common nettle (*Urtica dioica*), “that everyone was looking for in Amsterdam,” according to one participant. Horse chestnuts (*Aesculus hippocastanum* L.) were “ground and sold as coffee powder, but had to be thrown away because it tasted completely rancid”. Dahlia bulbs (*Dahlia* spp*.*) were consumed, although they had “no caloric value, but did give a satisfactory feeling”. The 16 most frequently mentioned species are listed in Table 3.

**Table 2.** List of all emergency food species mentioned during the 78 interviews

Scientific name	Vernacular names	Family	Citation frequency	Domestication status	Parts used	Mode of consumption
*Beta vulgaris* L. subsp. *vulgaris* var. *altissima*	Sugar beet (en), suikerbiet (du)	Amaranthaceae	66 (85%)	Cultivated	Root	Raw Boiled Pancakes Juice Birthday cake Bread Nasi goreng Foam
*Tulipa* spp. L.	Tulip (en), tulp (du)	Liliaceae	49 (59%)	Cultivated	Bulb	Boiled Mash Bread Soup
*Solanum tuberosum* L.	Potato (en), aardappel (du)	Solanaceae	35 (45%)	Cultivated	Tuber skin	Soup Dried; used as fuel
*Cichorium intybus* L. var. *sativum* ^a^	Chicory (en), cichorei (du)	Asteraceae	15 (19%)	Cultivated^b^	Root	Dried and ground to coffee powder
*Urtica dioica* L.	Common nettle (en), brandnetel (du)	Urticaceae	13 (17%)	Wild	Leaves	Soup Put in mash Sautéed
*Rubus* ssp*.* L.	Blackberry (en), braam (du)	Rosaceae	12 (15%)	Wild	Fruit	Raw Jam
*Fagus sylvatica* L.	Beech (en), beuk (du)	Fagaceae	9 (12%)	Wild	Nut	Raw Baked
*Raphanus sativus* L.	Radish (en), radijs (du)	Brassicaceae	7 (9%)	Cultivated	Leaves	Put in mash Soup
*Rosa* ssp*.*	Rose (en), roos (du)	Rosaceae	7 (9%)	Wild	Fruit	Raw Jam
*Juglans regia* L.	English walnut (en), walnoot (du)	Juglandaceae	6 (8%)	Wild	Seed	Raw Roasted
*Castanea sativa* Mill.	Sweet chestnut (en), tamme kastanje (du)	Fagaceae	6 (8%)	Wild	Seed	Roasted
*Rumex* ssp*.*	Sorrel (en), zuring (du)	Polygonaceae	6 (8%)	Wild	Leaves	Soup Sautéed
*Taraxacum officinale* L.	Common dandelion (en), paardenbloem (du)	Asteraceae	5 (6%)	Wild	Leaves	Raw Sautéed
*Daucus carota* L. ssp. *sativus*	Carrot (en), wortel (du)	Apiaceae	4 (5%)	Cultivated	Leaves	Put in mash Sautéed
*Beta vulgaris* L. ssp. *vulgaris* var. *crassa*	Fodder beet (en), voederbiet (du)	Brassicaceae	4 (5%)	Cultivated	Root	Cooking
*Brassica oleracea* L. convar*. oleracea* var*. gemmifera*	Brussel sprouts (en), spruitkool (du)	Brassicaceae	4 (5%)	Cultivated	Leaves and stems	Boiled
*Dahlia* sp.	Dahlia (en), dahlia (du)	Asteraceae	3 (4%)	Cultivated	Roots	Boiled
*Gladiolus* sp.	Gladiolus (en), gladiool (du)	Iridaceae	3 (4%)	Cultivated	Bulb	Boiled
^a^ *Petasites hybridus* ( L.) G.Gaertn., B.Mey. & Scherb.	Butterbur (en), groot hoefblad (du)	Asteraceae	3 (4%)	Wild	Leaves	Dried and smoked
^a^	Flower bulbs (en), bloembollen (du)	–	3 (4%)	Cultivated	Bulbs	Cooking
^a^	Mushrooms (en), paddenstoelen (du)	–	2 (3%)	Wild	Fruiting body	Baking
*Zea mays* subsp. *mays* L.	Fodder maize (en), voedermais (du)	Poaceae	2 (3%)	Cultivated	Kernel	Boiled
*Crocus* ssp*.*	Crocus (en), krokus (du)	Iridaceae	2 (3%)	Cultivated	Bulb	Boiled
*Galium odoratum* (L.) Scop.	Sweetcented woodruff (en), lievevrouwebedstro (du)	Rubiaceae	2 (3%)	Wild	Leaves	Put in wine
*Quercus robur* L.	English oak (en), zomereik (du)	Fagaceae	2 (3%)	Wild	Nut	Roasted
*Claytonia perfoliata* Donn ex Willd.	Winter purslane (en), winterpostelein (du)	Portulacaceae	2 (3%)	Wild	Leaves	Vegetable
*Aesculus hippocastanum* L.	Horse chestnut (en), witte paardenkastanje (du)	Sapindaceae	2 (3%)	Wild	Seed	Roasted and ground into coffee powder
*Trifolium* ssp.	Clover (en), klaver (du)	Fabaceae	2 (3%)	Wild	Leaves	Soup
*Aegopodium podagraria* L.	ground elder (en), zevenblad (du)	Apiaceae	2 (3%)	Wild	Foliage	Sautéed Soup
*Cantharellus cibarius* Fr.	Chanterelle (en), cantharel (du)	Cantharellacea	2 (3%)	Wild	Fruiting body	–
*Agaricus campestris* L.	Field mushroom (en), gewone weidechampignon (du)	Agaricaceae	2 (3%)	Wild	Fruiting body	–
*Solanum tuberosum* L.	Potato (fodder) (en), aardappel (veevoer) (du)	Solanaceae	2 (3%)	Cultivated	Tuber	Boiled
*Sambucus nigra* L.	Black elder (en), vlier (du)	Adoxaceae	2 (3%)	Wild	Fruit and flowers	Juice from berries Cooking berries
*Iris* ssp*.*	Iris (en), iris (du)	Iridaceae	1 (1%)	Cultivated	Rhizome	–
*Typha latifolia* L.	Broadleaf cattail (en), lisdodde (du)	Typhaceae	1 (1%)	Wild	Roots and leaves	–
*Malus baccata* (L.) Borkh.	Wild apple (en), kersappel (du)	Rosaceae	1 (1%)	Cultivated	Fruit	Jam
*Vaccinium myrtillus* L.	European blueberry (en), bosbes (du)	Ericaceae	1 (1%)	Wild	Fruit	–
*Boletus edulis* Bull.	Porcini (en), eekhoorntjesbrood (du)	Boletaceae	1 (1%)	Wild	Fruiting body	–
*Rubus idaeus* L.	Raspberry (en), framboos (du)	Rosaceae	1 (1%)	Wild	Leaves	Tea
*Rosa rubiginosa* L.	Sweet briar (en), egelantier (du)	Rosaceae	1 (1%)	Wild	Leaves	Tea
*Bellis perennis* L.	Common daisy (en), madeliefje (du)	Asteraceae	1 (1%)	Wild	Leaves, flowers	–
*Stellaria media* (L.) Vill.^a^ (NC)	Commom chickweed (en), vogelmuur (du)	Caryophyllaceae	1 (1%)	Wild	Whole plant	Salad

^a^Not included in statistical analysis

^b^Origin could not be determined with certainty

NC Not confirmed. The species is based on the description made by the interviewed person, but the name of the plant was not mentioned

*en* English, *du* Dutch

**Table 3:** The 16 most frequently mentioned famine food species during the 78 interviews, drawn from the combined data of the free-listing and the checks with the preliminary list of famine food species

Scientific name	Common name (English)	Parts used	Citation frequency [%]
*Beta vulgaris* ssp. *vulgaris* var. *altissima*	Sugar beet	Tuber	85
*Tulipa* spp.	Tulip	Bulb	59
*Solanum tuberosum*	Potato	Tuber peel	45
*Cichorium intybus* var. *sativum* ^a^	Chicory	Root	19
*Urtica dioica*	Common nettle	Leaves	17
*Rubus* spp*.* ^b^	Blackberry	Fruit	15
*Fagus sylvatica*	Beech	Nut	12
*Rosa* spp*.* ^b^	Rose	Fruit	9
*Raphanus sativus*	Radish	Foliage	9
*Juglans regia*	English walnut	Seed	8
*Castanea sativa*	Sweet chestnut	Seed	8
*Rumex* spp*.* ^b^	Sorrel	Leaves	8
*Taraxacum officinale*	Common dandelion	Leaves	6
*Daucus carota* subsp. *sativum*	Carrot	Leaves	5
*Brassica oleracea* convar*. oleracea* var*. gemmifera*	Brussel sprouts (fodder)	Leaves, stems	5
*Beta vulgaris* subsp*. vulgaris* var*. crassa*	Fodder beet	Tuber	5

^a^Excluded from our statistical analysis because it was unclear whether this coffee substitute was of wild or cultivated origin

^b^Probably more than one species in this genus was consumed

On average, 3.4 emergency food species were mentioned during the interviews. The number of species ranged from 1 to 10. Not all species from the written sources listed in Table 1 (wartime cookbooks, leaflets, or pamphlets) were mentioned during the interviews. Whether our participants (or their families) had not consumed them or whether they had forgotten them or were unaware of this because of their young age during the war can no longer be traced. The war survivors mentioned to have consumed 27 of the 45 species or varieties described in the cookbooks and literature. A total of 18 species or specific plant parts were only listed in written sources but not reported by our respondents. However, an additional 11 species were mentioned during our interviews but could not be traced as being consumed as emergency food in the literature.

When we analyzed the results of the free-listing exercise only (Table 4), the three most frequently mentioned famine species were the same as in the combined dataset (Table 3). Sugar beet had a by far the highest salience index, followed from a distance to tulip bulbs and the by potato peels and stinging nettles.

**Table 4:** Most frequently mentioned famine food species during the free-listing exercise, weighed by their frequency and mean position according to the Cognitive Salience Index [65]

Scientific name	*F*	OR	mP	*S*	NR
*Beta vulgaris* ssp. *vulgaris* var. *altissima*	47	1	1.489	0.406	1
*Tulipa* ssp*.*	24	2	1.625	0.189	2
*Solanum tuberosum* (peels)	7	3	1.714	0.052	3
*Urtica dioica*	4	4	1	0.051	4
*Rumex acetosa*	2	8.5	1	0.026	5.5
*Cantharellus cibarius*	2	8.5	1	0.0261	5.5
*Agaricus campestris*	2	8.5	1.5	0.017	7
*Brassica oleracea* convar. *oleracea* var. *gemmifera*	3	5.5	2.333	0.016	8
*Rubus fruticosus*	2	8.5	2	0.013	9.5
*Fagus sylvatica*	3	5.5	3	0.013	9.5

*F* frequency, *OR* old rank based on frequency, *mP* mean position of mentioning, *S* salience index, *NR* new rank with weighed position

**Fig. 4:**
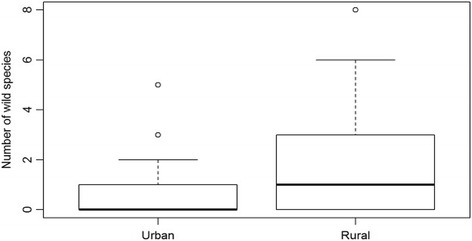
Boxplot showing the number of wild species consumed by urban and rural respondents. Differences are significant (*p* = 0.0087)

**Fig. 5:**
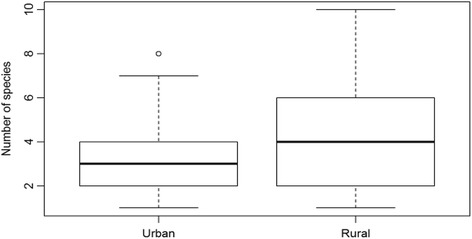
Boxplot showing the total number of species (cultivated and wild) consumed by rural (*n* = 52) and urban respondents (*n* = 26). Differences are significant (*p* = 0.0099)

**Fig. 6:**
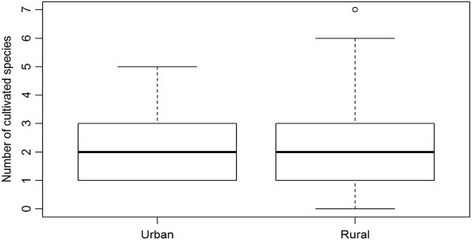
Boxplot showing the number of cultivated species consumed by urban (*n* = 52) and rural respondents (*n* = 26). No significant difference was found (*p* = 0.4582)

The data of all groups indeed had unequal variances (Table 5). Rural people consumed higher numbers of wild species than urban people (Fig. 4). War survivors in rural areas ate significantly more famine food species than people in urban areas (Fig. 5). No significant difference was found between the number of cultivated famine food species consumed by urban and rural people (Fig. 6).

**Table 5:** Total number of famine food species consumed by participants from urban and rural areas

	Urban (*n* = 52)	Rural (*n* = 26)	*t* value	*p* value	Power
No. of wild species	0.6 ± 1.14^a^	2.0 ± 2.76	− 2.5204	0.0087	0.93
No. of cultivated species	2.3 ± 1.06	2.3 ± 1.70	− 0.1058	0.4582^b^	0.08
No. of total species	2.9 ± 1.66	4.4 ± 2.82	− 2.4443	0.0099	1.00

^a^All values represent means ± standard deviations

^b^No significant differences

Only three of the 78 respondents were familiar with wartime cookbooks and pamphlets. Another three (relatives of) war survivors learned about the possibility to consume products like tulip bulbs and sugar beets from newspaper articles. The majority of the participants said they or their families knew what to eat from other people in their surroundings: “everybody suddenly knew that products like tulip bulbs were edible.”

When war survivors were asked about any illness or adverse effects due to eating certain famine food species, the majority (76%) could not recall this anymore. The few ailments resulting from eating famine food were sore throats from the consumption of (raw) sugar beets (*n* = 8) and tulip bulbs (*n* = 3). Three participants also reported stomachache after eating sugar beets and tulip bulbs. Two persons remembered that the bad quality food from the soup kitchen made them vomit. Nearly all participants explicitly expressed negative opinions about the food served in soup kitchens, calling it “revolting,” “utterly disgusting,” or “making people sick.” Several persons said that even long after the war, the scent of the sugar beet processing plants in the Dutch countryside still made them nauseous, as it reminded them of the war. Although we did not explicitly ask for it, seven participants (9%) said their family possessed a garden in which they grew their own food. The rapid transformation of land into family vegetable plots is also described in literature [37]. Several participants mentioned that they or their family members had stolen food.

Not only vegetables and starch crops were scarce: luxury goods like coffee, tea, and tobacco were also unavailable for normal prices. People’s ingenuity was not limited to primary food sources, as they also substituted coffee, tobacco, and tea by surrogates of natural origin. Chicory root was mentioned 15 times as a common coffee substitute during the war. The root was roasted and ground to make a powder that could be used to brew coffee. Leaves of blackberry (*Rubus* spp*.*) were one of the many species served as a tea replacement. The large leaves of butterbur (*Petasites hybridus* (L.) G.Gaertn., B.Mey. & Scherb) were dried and smoked to substitute tobacco.

## Discussion

### Limitations of this research

We are fully aware that people’s memories about the Second World War have faded 71 years after date. The extensive time period between the war and the moment our interviews took place means that we certainly have not captured all famine food species consumed by our participants. They were chiefly quite young at the time of liberation, so our results partly rely on the information they received from parents, older siblings, other family members, acquaintances, or friends. Still, our participants brought up detailed memories of their own experiences and the stories told by their relatives. The repetitive encounter of statements in the interviews provided a solid base for their validity. Archival data further supported the evidence of the consumption of certain species as famine foods. The generation of our participants is the last to have first-hand experiences of World War II, and therefore, our research can be considered as a case of “salvage ethnobotany,” described as “recovering plant knowledge that otherwise might be lost” [2]. We generally encountered eagerness among our elder participants to share their memories on the war. Perhaps the focus on food and plants, instead of personal tragedies, made the sensitive subject easier to share.

### Suggestions for further research

We were approached by several survivors of Japanese internment camps in the former Dutch colony of Indonesia. They told us that they remembered eating grasshoppers, raw *Capsicum* peppers, waste material, and wild plants. Although we limited our research to the Netherlands, famine foods consumed during the Japanese occupation in Indonesia may have been documented in published diaries [67] but have never been examined from an ethnobotanical perspective. As fewer survivors are left every year, there is an urgent need for specific research on this subject, with additional fieldwork in Indonesia to verify the identifications of local plant names.

Although not included in our interviews, several participants mentioned to have caught and eaten wild animals and sacrificed their pets for consumption. Two persons ate cat meat, which reminded them of rabbit in flavor. A woman told about her father making a cat-trap from a barrel dug into the ground with a wooden board put over it. A piece of rope with a dead mouse on the end was attached to this board so it would dangle in the middle of the barrel. When a cat reached for the bait it would fall in. She said her family “collected many cats this way.” A man supposedly ate dog after his family bought a piece of meat from the local store. He reported that the meat tasted “horrible” and that “after that day the dog of the store owner was suddenly gone.” Other reported eaten animals are pheasants, earthworms, and house sparrows. In all the major cities, war survivors talked about the cutting of trees, “until there were no more trees left standing.” Some people were sent out as kids to collect coals and timber. One interviewee said he “sneaked onto the train yard to look for still usable coals in piles of used train fuel.” There is still a wealth of information available in these personal childhood memories on animal and fuel collection during World War II.

### Sugar beets and flower bulbs

**Fig. 7:**
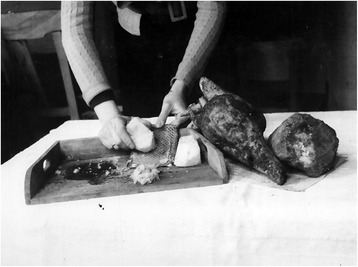
Woman grating sugar beets during the war. Source: http://www.brandgrens.nl/hongerwinter

Sugar beet was by far the most frequently consumed famine food species during World War II (Fig. 7). This is not surprising, given the fact that this crop was included in the ration distributed by the government and processed into soup kitchen meals [32]. Some stores sold foam made of whisked water sweetened with sugar beet [68]. A brother and sister who remembered eating this foam said that “you had to eat it really quick, otherwise it would completely dissolve in your hands.” One respondent remembered a recipe for a birthday cake made out of sugar beets, “which surprisingly tasted like the real thing.” Tulip bulbs, which had been actively promoted as edible, also formed a considerable amount of extra nutrition in the wartime diet of the Dutch. The high number of ornamental species consumed as famine food is explained by the importance of the Netherlands in the ornamental flower trade [69]. The export of tulip bulbs dropped to almost zero during the war. The large surplus of bulbs that could not be sold stimulated the government to promote it actively in newspapers and public restaurants. As a result of their wide availability, flower bulbs were among the most consumed wartime foods [68].

The stomachache caused by the consumption of tulip bulbs, as reported during the interviews, can be explained by their poisonous compounds. Despite the fact that tulip bulbs were officially confirmed to be suitable for human consumption by a Dutch doctor in 1944, they contain amounts of a DNA-synthesis inhibiting protein named tulipin [46, 70]. Toxicity was often given as a reason by our participants to explain why they or their relatives did not collect wild plants or mushrooms. Some native, edible Dutch wild fungi strongly resemble toxic species, so this fear of poisoning is well placed [71]. Although none of the participants experienced a fatality in their surroundings, evidence for poisoning during the wartime period is found in toxicological reports of patients caused by the consumption of hyacinth bulbs and high quantities of beechnuts [72, 73]. It is also possible that fatal accidents occurred with poisonous *Narcissus* bulbs, considering that they closely resemble other edible bulbs [38].

**Fig. 8:**
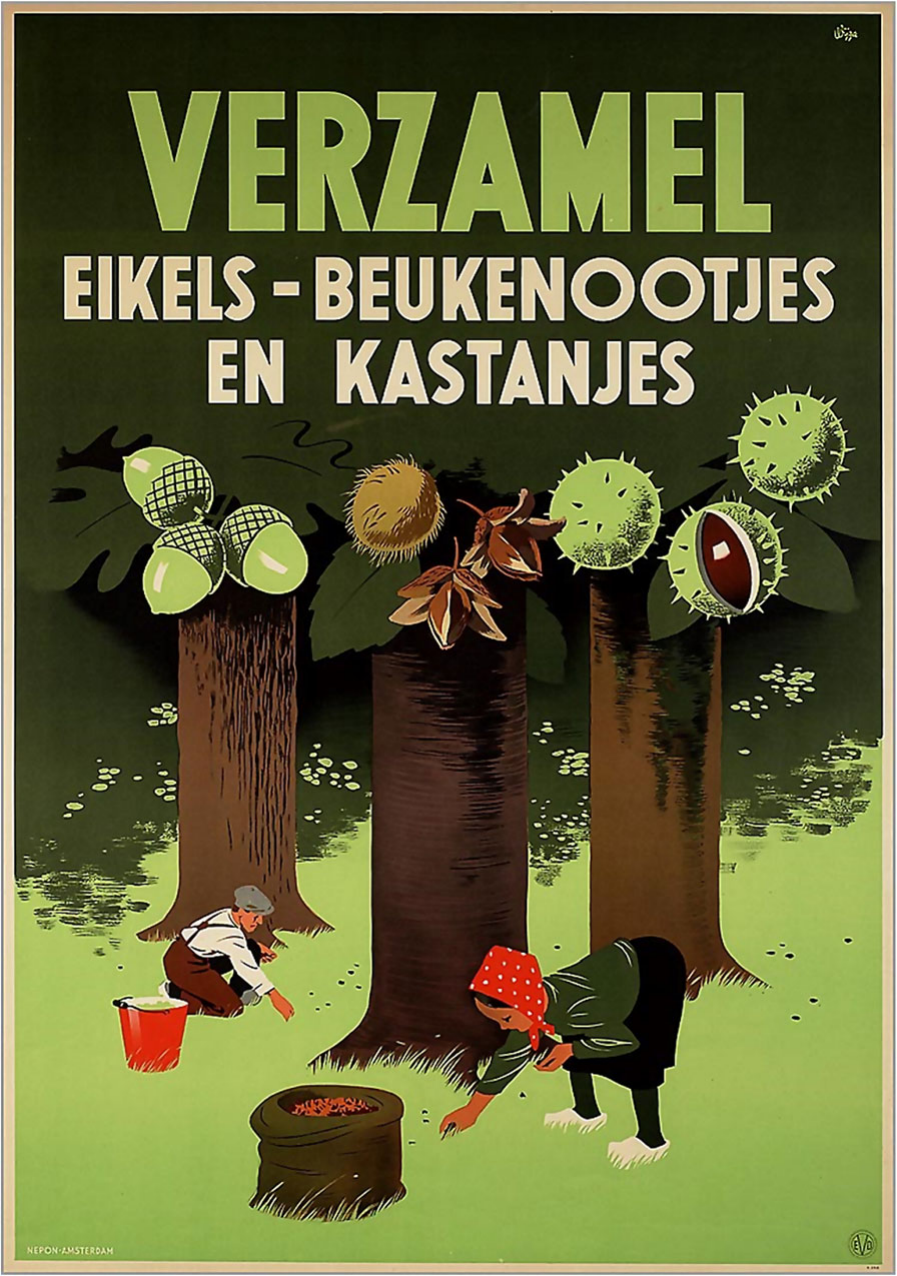
Pamphlet to encourage people to collect acorns and beech- and chestnuts. Source: [86]

The wartime cookbooks (Fig. 8) were not used as widespread as anticipated. Although recipes for preparing tulip bulbs and sugar beets did reach people, most ethnobotanical information on edible species and preparation methods was passed orally within families and among neighbors. Some participants said the edibility of sugar beets and tulip bulbs became common knowledge at a certain point. However, as most participants were not old enough to read or cook with famine food species themselves, the actual use of this written information may have been higher than our data suggest.

A logical explanation for the higher number of consumed wild species can be that people who live in rural areas are more frequently exposed to wild plants and therefore more conscious about their edibility [74]. Being surrounded by famine food species repeatedly later in life reminds people of their use during the war, keeping their memory alive.

### Consumption of famine food after the war

Lately, a revival of wild collection has taken place in Western Europe [7]. The increasing aversion towards processed foods has caused a rise in self-grown and wild-collected plant foods [75, 76]. Foraging in the Netherlands, however, is not as common as in other European countries like Poland, Croatia, Italy, and Belarus [39, 41], where knowledge on wild food collection is still passed on from generation to generation. This tradition vanished early on from the Netherlands, probably due to the relatively small areas with natural vegetation in the Netherlands, the high population density, urbanization, and relative wealth, compared to elsewhere in Europe [63, 77, 78].

While in Western Europe the last serious food shortages occurred during the Second World War, hunger still posed a serious problem in the Balkan from 1992 to 1995. The 3-year siege of the city of Sarajevo during the Bosnian War caused an outbreak of massive famine. The Bosnian botanist Sulejman Redžić educated his fellow citizens on wild plant utility in a similar manner as the Dutch government did in the 1940s [79, 80]. The three most consumed mushrooms found in his research are the same three species as appear in our data. The Yugoslavian military had already done extensive research in the 1960s on famine food consumption during WWI and WWII with the aim to use this knowledge on wild edible plants and animals for survival training of their army [39]. One of our participants in the Netherlands, a retired botanist, was also asked to use his knowledge on wartime wild plant consumption to give a training to Dutch soldiers in the 1950s.

The economic crisis of 2007–2009 in Greece triggered a comeback of wild plant foraging among impoverished Greeks [81]. A book containing “starvation recipes” published by Greek newspapers during World War II called the attention of money-strapped Greeks [82]. Outside Europe, life-threatening starvation is still going on today, as reports of Syrian citizens eating grass and wild plants during the siege of Aleppo [83]. Hunger is as old as humanity itself and will most likely not become a thing of the past. Knowledge on famine food species still contributes to people’s survival, today and in the future.

## Conclusion

Our research shows that the once crucial knowledge on wild edible plants and famine food sources is still present among elder Dutch citizens. Even after 71 years, this knowledge has not yet disappeared from the Dutch society and probably will not vanish anymore with the current widespread access to information on wild food sources. Plant identification, however, is still a skill that has to be taught in practice. The information on famine food sources supplied by several institutions was not distributed widely. For the necessary revival of this knowledge during the 1940s, people needed to consult a small group of mostly elderly people who still had the know-how. Inhabitants of rural areas listed more wild-collected plants than people who had spent the war in cities, while the number of cultivated famine food species they consumed during World War II did not differ. Rural people consumed more famine food altogether than urban citizens. Apart from some written reports on poisoning by wild-collected food or ornamental bulbs, our participants did not remember major complications caused by the consumption of famine foods, as long as they were well prepared or at least the hunger greatly overruled the possible uneasiness caused by them.

## Abbreviations

L: Naturali Biodiversity Center herbarium, Leiden, the Netherlands
NIOD: Dutch National Institute of War Documentation
WW II: Second World War (1940–1945)
